# Does Fragment-Specific Fixation Provide Better Functional Outcomes Following Trimalleolar Ankle Fractures?

**DOI:** 10.1177/19386400251343745

**Published:** 2025-06-19

**Authors:** Danuksha K Amarasena, Upamanyu Nath, Abhirun Das, Thomas Collins, Anand Pillai

**Affiliations:** The University of Manchester and University Hospitals of North Midlands, Manchester, UK; Manchester University NHS Foundation Trust, Manchester, UK; Manchester University NHS Foundation Trust, Manchester, UK; Manchester University NHS Foundation Trust, Manchester, UK; Manchester University NHS Foundation Trust, Manchester, UK

**Keywords:** ankle fractures, trimalleolar fracture, fragment-specific fixation, volition, orthopaedic surgery, foot and ankle

## Abstract

**Background:**

Fractures to the ankle account for nearly 10% of all fractures. The trimalleolar configuration of ankle fracture is a notoriously unstable injury encompassing injury to the medial, lateral, and posterior malleolus, often occurring as a result of rotational force to the ankle. Historically, poorer outcomes have been associated with fractures of the posterior malleolar component, which were broadly categorized as a single homogenous group. Recent advancements and greater appreciation of fracture pathomechanisms have aided fragment classification and hence tailored fixation. Aims. Our study compares the post-operative functional outcomes following novel fragment-specific plating (Volition) against conventional plating during the surgical fixation of trimalleolar ankle fractures. Post-operative functional outcomes were measured at 12 months using the Foot and Ankle Disability Index (FADI) and Manchester-Oxford Foot Questionnaire (MOXFQ) as patient-reported outcome measures (PROMs).

**Methods:**

We conducted a retrospective cohort study of patients admitted to our orthopaedic department for a corrective surgery following a trimalleolar ankle fracture. Each fracture was confirmed via computed tomography (CT) imaging and corrected using either fragment-specific or conventional plating. Post-operatively, patients were followed up functionally and radiologically.

**Results:**

Our study included 22 matched pairs of patients, each of who required surgical fixation for a trimalleolar ankle fracture. The FADI and MOXFQ questionnaires were conducted to assess functional outcomes during the post-operative period. The PROM data indicated that there were statistically significant superior outcomes in both the activity and pain subscales of the FADI (P > .05). However, no significant differences were observed in the MOXFQ scores.

**Conclusion:**

This study concludes that fragment-specific plating of the posterior malleolar component provides superior functional outcomes in terms of pain and activity levels following trimalleolar ankle fractures, as measured by the FADI. Larger studies with longer follow-up are needed to confirm these results and guide standardized treatment protocols.

**Level of Evidence:**

Level 2

“The trimalleolar configuration of ankle fracture is a notoriously unstable injury encompassing injury to the medial, lateral, and posterior malleolus, often occurring as a result of rotational force to the ankle.”

## Introduction

The ankle joint is a complex joint comprised of an intricate network of ligaments working cohesively to maintain its stability. Despite this, ankle fractures are one of the most common types of lower limb injuries presenting to orthopaedic surgeons and account for almost 10% of all fractures.^1,2^

Trimalleolar fracture refers to a fracture involving a combination of the medial, lateral, and, most importantly, the posterior malleolus (an anatomical protuberance situated posteriorly on the tibial plafond).^3^ These fractures occur as a result of rotational force to the ankle, where external rotation of the talus causes avulsion by the posterior inferior tibiofibular ligament, sometimes combined with axial loading to produce larger posterior tibial margin fractures.^4^

Owing to the complex configuration of the ankle joint, trimalleolar ankle fractures often result in concomitant injury to surrounding ligaments, leading to compromised stability of the joint.^5^ The management of these ankle fractures is guided by the stability of this joint, and so accurate diagnosis and classification are imperative to guiding management, which often require surgical intervention.^6,7^

Various approaches have been accepted to correct the structure of the posterior malleolar component of the trimalleolar ankle fracture with the hope of restoring stability and function following injury; however, there is no clear consensus on the optimal method of correction.^8^

The Volition (Ortho Solutions UK Ltd, Essex, UK) plating system has been developed specifically to address the known posterior malleolar fracture configurations, which optimizes the stabilization and aids restoration of articular congruency. Our study aims to compare the post-operative functional outcomes of complex trimalleolar ankle fractures treated with novel fragment-specific treatment using the Volition plating system against conventional plating systems. We measured functional outcomes through the use of patient-reported outcome measures (PROMs) including the Foot and Ankle Disability Index (FADI) and the Manchester-Oxford Foot Questionnaire (MOXFQ).

## Methods

We conducted a retrospective cohort review of skeletally mature patients admitted to our multidisciplinary team unit following a trimalleolar ankle fracture requiring corrective surgery. Patients reviewed in this study were admitted between October 2021 and February 2024. Fractures were confirmed and classified using computed tomography (CT) imaging with 3D reconstruction alongside axial, sagittal, and coronal plane views. Classification of fractures was done using the Mason and Molloy classification into type 1, 2a, 2b, and 3 fractures. Our exclusion criteria were patients less than 18 years of age and those who were involved in major trauma resulting in other injuries.

**Figure 1.**
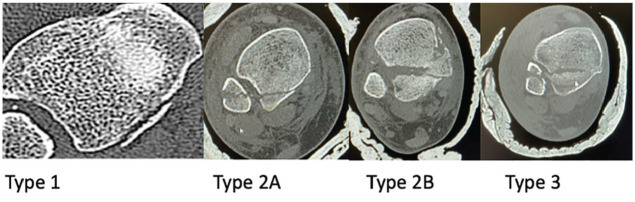
Axial CT scan showing different types of posterior malleolar fractures based on the Mason and Molloy classification.

**Figure 2.**
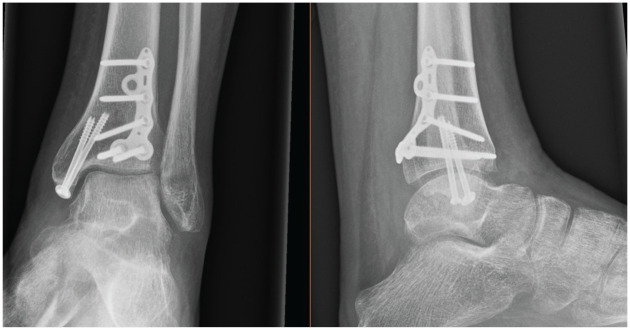
Radiograph showing (Volition) type IIA (posterolateral tibial plate) in situ.

**Figure 3.**
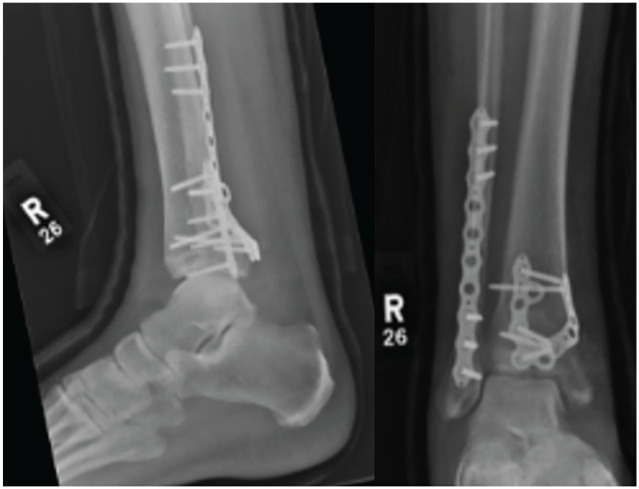
Radiograph showing (Volition) type IIB (posteromedial tibial plate) in situ.

**Figure 4.**
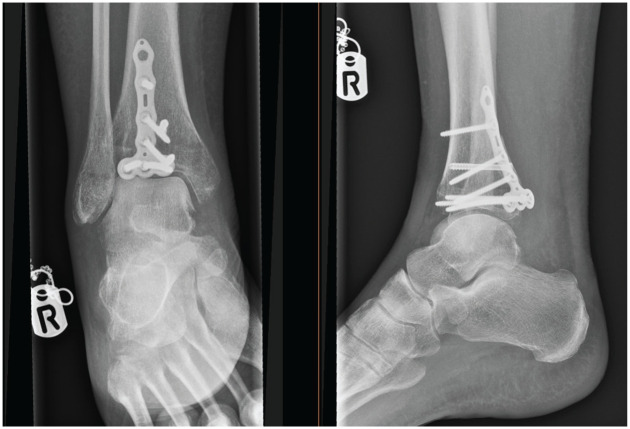
Radiograph showing (Volition) type III (posterior tibial plate) in situ.

Injuries were assessed for surrounding soft tissue concern prior to definitive correction. In instances where soft tissue was of concern or fractures were too unstable to be maintained in position with a backslab, an external fixation device was temporarily used until adequate resolution was achieved. Definitive surgical fixation was conducted using either fragment-specific implants (Volition) plates (Figures 1–4) or standard non–fragment-specific implants (“Non-Volition”).

Post-operatively, patients underwent consistent follow-up at 2, 6, 12, and 26 weeks. Assessment for evidence of clinical complications such as stiffness, pain, metalwork irritation, infection, nerve damage, complex regional pain syndrome (CRPS), or failure of union were conducted. Post-operative imaging was also conducted to confirm the adequacy of fixation and radiological union. At 12 months post procedure, each patient was evaluated for functional outcomes using PROMs. The PROMs incorporated in our study were the FADI and the MOXFQ.

The FADI is a validated questionnaire comprising 34 questions each answered on a 5-point Likert scale from 0 to 4.^9,10^ The questions are then grouped into 2 separate subscales being the activity subscale (where 0 represents “unable to do” and 4 represents “no difficulty at all”) and the pain subscale (where 0 means “unbearable pain” and 4 means “no pain”).^9,10^ As a result, a higher cumulative score in the FADI correlates to a better functional capability.

The MOXFQ is a validated questionnaire comprising 16 questions, which are also answered on a 5-point Likert scale.^10^ Each question is scored from 0 (representing the least severe) to 4 (representing the most severe). The questions are then grouped into 3 separate subscales representing the domains of walking/standing (7 questions), social interaction (4 questions), and pain (5 questions).^11^ As a result, a lower cumulative score in the MOXFQ correlates to a better functional capability. The grouped domains have been proved to have excellent validity, reliability, and responsiveness.

All patients meeting the cohort inclusion criteria were then subdivided into 2 groups determined by the method of fixation used. The first group consisted of those who had fractures corrected using fragment-specific plating (Volition), whereas the second group were those who underwent correction with non–fragment-specific (Non-Volition) plating. These patients were then matched into corresponding pairs prior to undergoing statistical analysis.

An Anderson-Darling normality test was performed, and the data were found to be non-parametric. The Wilcoxon Signed Rank Sum test was therefore used to determine if the differences observed between the Volition and Non-Volition PROM scores were statistically significant.

## Results

Our cohort consisted of 69 patients who had undergone surgical correction for a trimalleolar ankle fracture. The Volition group consisted of 35 patients, and the Non-Volition group consisted of the remaining 34 patients.

We then matched patients using the following parameters: age ±10 years, sex, and fracture classification. This constituted a total of 22 matched pairs and of whom statistical analysis was subsequently conducted. Analysis of post-operative radiographs confirmed that radiological union was successfully achieved by all patients with the mean time to union being 7.8 weeks in the Volition group compared to 8.8 weeks in the Non-Volition group. Further demographic comparisons among the groups are outlined in Table 1.

**Table 1.** Summary of demographic data.

Patient data	Volition implants	Non-Volition implants
Average age (years ± standard deviation)	49.7 ± 15.6	50.0 ± 15.1
Sex		
Male	8 (36.4%)	8 (36.4%)
Female	14 (63.6%)	14 (63.6%)
Side		
Left	6 (27.3%)	10 (45.5%)
Right	16 (72.7%)	11 (50.0%)
Bilateral	0 (0.0%)	1 (4.5%)
Average time to radiological union (weeks)	7.8	8.8

The summary table (Table 2) contains the 22 matched pairs comparing the outcome of Volition vs Non-Volition. The PROM data were collected at the final follow-up, which was 12 months post-operation. Our results demonstrate that Volition plating achieved superior outcome scores on average in both the FADI (indicated by a higher score) and MOXFQ (indicated by a lower score). These differences were found to be statistically significant (*P* > .05) in both the FADI activity and pain subscales; however, they were found not to be significant with regard to the MOXFQ (Table 2).

**Table 2.** Table comparing the PROM outcomes.

PROM	Volition implants (Average ± St Dev)	Non-Volition implants (Average ± St Dev)	Difference in PROM scores	*P*
MOXFQ pain	6.5 ± 4.1	9.7 ± 6.0	-3.3	.105
MOXFQ walking/standing	9.8 ± 9.1	14.7 ± 9.3	-4.9	.102
MOXFQ social interaction	4.7 ± 4.7	7.1 ± 5.6	-2.4	.119
FADI activity subscale	66.1 ± 20.9	33.6 ± 23.4	32.5	< .001
FADI pain subscale	12.5 ± 3.0	8.2 ± 4.8	4.4	.003

## Discussion

The management of ankle fractures has evolved in recent years, with preferred treatment now based on the fracture morphology rather than fragment size. The surgical treatment of complex ankle fractures, such as those involving the posterior malleolus, requires a comprehensive understanding of the injury mechanism and associated fracture pattern. In the literature, poor outcomes have traditionally been associated with posterior malleolar fractures that have been broadly categorized as a single homogenous group.^12,13^ However, more recently, the advancing understanding of fracture pathomechanisms has led to fragment-specific classification and treatment, where predictable posterior malleolar fracture patterns and the corresponding significance within the overall injury have been characterized.^12^

Despite there being a stable increase in the literature regarding the fixation of trimalleolar ankle fractures, there is yet to be a unified consensus on how best to treat these complex injuries. Conventional ankle fracture plating systems provide fixation based on the traditional single-group posterior malleolar characterization. In general, these generic plates do not adequately recognize the variable posterior components of trimalleolar fractures, which may lead to suboptimal outcomes. Conversely, the Volition Plating System has been developed specifically to address the known fracture patterns via fragment-specific fixation. The Volition plates are anatomically pre-contoured to optimize the reduction and stabilization of each fracture fragment, which may assist in the restoration of the articular congruency of the distal tibia and the stability of the ankle.

While fragment-specific fixation is a relatively novel approach to managing the complex trimalleolar ankle fracture, its use in the management in distal radius fractures of the hand is well documented in the literature. The use of the fragment-specific approaches not just accepted as a reasonable alternative to standard approaches but has been shown to produce excellent functional outcomes during the post-operative period.^14^

Our findings demonstrated statistically significant improvements in FADI scores, specifically in both the pain and activity subscales, for patients treated with the Volition system. However, no statistically significant difference was observed in MOXFQ scores between the 2 groups.

The difference in significance between these 2 PROMs may be attributed to the specific domains each tool measures. The FADI focuses more on functional activity and pain during movement, while the MOXFQ incorporates broader aspects such as social interaction and overall foot-related quality of life. It is possible that the anatomical contouring and stability provided by fragment-specific fixation led to improved biomechanical function, which was more sensitively captured by the FADI. However, factors such as post-operative rehabilitation, pre-existing comorbidities, or patient perception may have diluted differences in the MOXFQ domains.

We also considered whether fracture classification played a role in the outcomes. Although we matched patients by fracture type using the Mason and Molloy classification, subtle differences in fracture morphology or comminution severity may still have impacted results. In addition, syndesmotic fixation, although not consistently recorded across all cases, may have influenced both joint congruency and long-term function. Future studies should aim to stratify patients based on the presence or absence of syndesmotic injury and whether or not fixation was performed.

While our study offers a valuable comparison between the functional outcomes following fragment-specific fixation compared to standard plating fixation, we do acknowledge a few limitations. The primary limitation was the cohort size of matched pairs in this study, which limited statistical power, particularly when assessing secondary outcome measures. Despite growing interest, the use of fragment-specific fixation is a relatively novel concept when treating trimalleolar ankle fractures and is not yet widely adopted in clinical practice, thereby limiting the eligible patient population. Having an increased cohort size would allow us to obtain greater numbers of matched pairs, leading to an increased power of our study and so achieving a more definitive conclusion. In addition, the limited follow-up duration and heterogeneity in fracture morphology and syndesmotic fixation within the cohort may have influenced outcomes. We plan to follow up with all patients at the 3-year post-operative mark to address these limitations and further strengthen our findings.

## Conclusion

Our study found that fragment-specific fixation of the posterior malleolar components using the Volition plating system resulted in significantly better functional outcomes in terms of pain and activity, as measured by the FADI, compared to conventional plating. These findings support fragment-specific fixation as a promising alternative; however, larger studies with longer follow-up are needed to confirm these results and guide standardized treatment protocols.
